# Dispersion of the Uterine Fibroids—Literature of the Profession—The Advice of Dr. Washington L. Atlee

**Published:** 1869-10

**Authors:** 


### Editorial Department.

### Dispersion of the Uterine Fibroids—Literature of the Profession—The Advice of Dr. Washington L. Atlee.

Dr. ATLEE, of Philadelphia, very kindly advises us to examine the literature of the profession upon the dispersion of Fibroid Tumors; and we will accept the suggestion, with the view that our readers may also like to know the opinions of the profession, Dr. ATLEE maintaining, dogmatically, that medication—muriate of ammonia—will sometimes disperse such growths. There is nothing in our profession more important for us to understand than every possible method of dispersing fibroid growths. If medication will do it, it is quite time our standard authors are aware of it. In a recent work upon Diseases of Women, by Prof. HODGE, we find on the 454th page, speaking of these fibrous tumors, the following paragraph. We read it with astonishment, since he is a neighbor of Dr. ATLEE's, and would be the more likely to know his experiences, and the general fact that medicine would sometimes disperse these growths. "Medicines, in these cases, are of no further use than to maintain the healthy functions of the organs of the economy, and to impart tone to the system; as resolvents, they are not to be trusted." It may be well to state in passing, that this author speaks of Dr. ATLEE's "boldly resorting to the scalpel, with all its immediate and remote dangers;" and does not seem ignorant of any important facts concerning fibroid tumors; and yet, strange enough, muriate of ammonia, is not mentioned at all—and operative interference is mainly, if not in all cases opposed.

Leaving Philadelphia, we will go down to Chicago and see if they sometimes disperse uterine fibroids with muriate of ammonia. Dr. BYFORD says: "These organized growths are engrafted upon the system, and are supported by the same processes of nutrition, that other parts of the body are sustained by; so that no special medical treatment exercises much influence over them." Says: "The profession are not agreed upon this point. A certain number of remedies are credited with curative effects upon almost all sorts of tumors, and among them uterine tumors. Iodine, I think, stands at the head of the list." He mentions mercury next—speaks of alkalies, soda and potassa, but does not evon mention muriate of ammonia—does not seem to know that it will disperse these growths. Says: "When it is remembered that these tumors disappear, so far as we can judge, spontaneously, and that they are interrupted in their growth by trifling and inappreciable mechanical causes, there will be room to doubt whether the above mentioned medicines have any effect upon them. I have not learned anything favorable to them in this respect."

The recent literature of the profession in Chicago points unfavorably both to dispersion and the ammonia; and we will pursue the inquiry to New York. Prof. T. GAILLARD THOMAS, M.D., in his recent work on the Diseases of Women, says:—"Whether their absorption can be excited by any medicines at our command, is very doubtful. Tumors have, in certain instances, been known to disappear while drugs have been employed, and perhaps they did so in consequence of their use. But no such effect can be looked for with any confidence. Indeed, with our present experience, such a result must be regarded as decidedly exceptional.

SCANZONI, after advising those medicines, [not mentioning muriate of ammonia] which are most popular as stimulants of absorption, says: "We do not remember a single case in which, with the means indicated, or with others, we have obtained the complete cure of a fibrous body; and if, in various quarters, fortunate cases of cure are cited, we must, if the tumor has really disappeared, doubt the accuracy of the diagnosis as to the fibrous nature of the malady. We even believe that it is not possible by therapeutic means, by baths, etc., to obtain a sensible diminution of a real fibrous tumor."

Dr. WEST speaks of Iodine and Mercury as having been recommended for causing absorption of fibroid tumors. He regards Iodine as promising most, and uses it with yearly lessening faith in its efficacy, "in a very large proportion of cases no effect whatever has appeared to follow its administration." Nothing said of muriate of ammonia.

GRAILY HEWETT, of London, says: "We know of no means whereby the formation of these tumors can be prevented, and when they are of large size, we know of no means whereby they can be made to disappear, short of a surgical operation."

We had supposed that we were tolerably well informed upon the subject of uterine fibroids—that our own personal experience was quite adequate for the formation of an intelligent opinion, and also, that we were quite familiar with the literature of the profession on this point, however, since examining recent authors by the advice of Dr. ATLEE, we wonder, a thousand times more, that he should gravely announce that such growths are sometimes dispersed by medication. If he really believes this, he is entitled to all due consideration, for doubtless he has had large experience in such diseases. He will, however, be generous enough, after noticing the statement of the above authors, to let those of us who have had abundant opportunities for observation, believe, if we must, with SCANZONI, and doubtless very many others, "that it is not possible, by therapeutic means, by baths, etc., (by muriate of ammonia, etc.,) to obtain a sensible diminution of a real fibrous tumor, especially if of large size, "of ten or twelve years growth, and weighing twelve or fifteen pounds."

As we cannot pursue our examination of this point further at present, we will hurriedly inquire into the therapeutical value of muriate of ammonia in this respect, though it will be noticed that no one of the authors quoted mention it. In Stille's Therapeutics and Materia Medica, we find it spoken of as having been *supposed* to be useful in very many diseases, as having been usefully substituted for Mercury and Iodine, and as having influence in various ways, and by a variety of modes of application, but we find nothing here or elsewhere, at all to the point so far as the dispersion of fibroid tumors is concerned. We were astonished at the assertion that fibrous tumors were sometimes dispersed by medication. Our surprise was increased by the suggestion that muriate of ammonia would do this, and we intimated our unbelief in sufficiently obvious terms. Dr. Atlee regards this as unworthy reply, and most graciously recommends us to the literature and ethics of the profession. Will any of our readers be surprised that we received Dr. Atlee's announcement with astonishment? two of the most *"remarkable"* propositions which could have been made in uterine surgery. We had partially recovered from the surprise, when in his communication in the present number, he repeats the sentiment, and intimates that the literature of the profession sustains him, or that, at least, it might do us some good to examine it.

If muriate of ammonia will disperse uterine fibroids, it is a good thing, and time the recent authors make some mention of it; it is an item of intelligence which may well be circulated in medical journals, and which we venture the opinion will pass as *news* with the great body of the profession. "Unworthy of notice." Thank you, sir: that is the opinion we entertained of the original propositions. It is quite unbecoming a reasonably well employed man, to spend time in discussing a subject upon which there is but one side, and consequently we made no "criticism"—simply said, the propositions contained in the letter were absurd, and gave them little notice—do not now think them worth notice, with all due regard, however, to the high professional character and standing of Dr. ATLEE.

